# Evaluation of stiffness and plastic deformation of active ceramic self-ligating bracket clips after repetitive opening and closure movements

**DOI:** 10.1590/2176-9451.20.4.045-050.oar

**Published:** 2015

**Authors:** Grace Kelly Martins Carneiro, Juliano Alves Roque, Aguinaldo Silva Garcez Segundo, Hideo Suzuki

**Affiliations:** 1 Master's student in Orthodontics, Faculdade e Centro de Pesquisas São Leopoldo Mandic, Campinas, São Paulo, Brazil; 2 Assistant professor, Faculdade e Centro de Pesquisas São Leopoldo Mandic, Department of Orthodontics, Campinas, São Paulo, Brazil; 3 Professor, Faculdade e Centro de Pesquisas São Leopoldo Mandic, Department of Orthodontics, Campinas, São Paulo, Brazil; 4 Head of the Master's program in Orthodontics, Faculdade e Centro de Pesquisas São Leopoldo Mandic, Department of Orthodontics, Campinas, São Paulo, Brazil

**Keywords:** Orthodontic brackets, Biomechanics, Fatigue, Laboratory automation

## Abstract

**OBJECTIVE:**

The aim of this study was to assess whether repetitive opening and closure of self-ligating bracket clips can cause plastic deformation of the clip.

**METHODS:**

Three types of active/interactive ceramic self-ligating brackets (n = 20) were tested: In-Ovation C, Quicklear and WOW. A standardized controlled device performed 500 cycles of opening and closure movements of the bracket clip with proper instruments and techniques adapted as recommended by the manufacturer of each bracket type. Two tensile tests, one before and one after the repetitive cycles, were performed to assess the stiffness of the clips. To this end, a custom-made stainless steel 0.40 x 0.40 mm wire was inserted into the bracket slot and adapted to the universal testing machine (EMIC DL2000), after which measurements were recorded. On the loading portion of the loading-unloading curve of clips, the slope fitted a first-degree equation curve to determine the stiffness/deflection rate of the clip.

**RESULTS:**

The results of plastic deformation showed no significant difference among bracket types before and after the 500 cycles of opening and closure (*p* = 0.811). There were significant differences on stiffness among the three types of brackets (*p* = 0.005). The WOW bracket had higher mean values, whereas Quicklear bracket had lower values, regardless of the opening/closure cycle.

**CONCLUSION:**

Repetitive controlled opening and closure movements of the clip did not alter stiffness or cause plastic deformation.

## Abstract (pt)

**OBJETIVO:**

o objetivo deste estudo foi avaliar se a abertura e o fechamento repetitivo do clipe de braquetes autoligáveis podem causar deformação plástica do clipe.

**MÉTODOS:**

três tipos de braquetes autoligáveis ativos/interativos estéticos (n = 20) foram testados: In-Ovation C, Quicklear e WOW. Um dispositivo realizou, de forma controlada e padronizada, 500 ciclos de abertura e de fechamento do clipe, com instrumentos e técnicas adequadas, tal como recomendado pelo fabricante de cada tipo de braquete. Dois ensaios de tração, um antes e um depois dos ciclos repetitivos, foram realizados para se avaliar a rigidez dos clipes. Para o ensaio de tração, um fio de 0,40 x 0,40mm de aço inoxidável foi inserido no *slot* do braquete e adaptado em uma máquina universal de ensaios (EMIC DL2000), sendo as medições registradas. Na porção retilínea da curva de carregamento dos clipes, a inclinação é definida por uma equação de primeiro grau, determinando o coeficiente de elasticidade (rigidez) do clipe.

**RESULTADOS:**

os resultados quanto à deformação plástica não mostraram nenhuma diferença significativa entre os tipos de braquetes, antes e depois dos 500 ciclos de abertura e de fechamento (p = 0,811). Foram encontradas diferenças significativas na rigidez entre os três tipos de braquetes (p = 0,005): o braquete WOW demonstrou valores médios mais elevados, e o braquete Quicklear demonstrou valores mais baixos, independentemente do ciclo de abertura e fechamento.

**CONCLUSÃO:**

movimentos repetitivos controlados de abertura e de fechamento do clipe não alteraram a rigidez ou causaram deformação plástica.

## INTRODUCTION

Self-ligating brackets are not a new concept, they were first developed in the 1930s. Since then, several types of self-ligating brackets have been commercially available. In general, the self-ligating system is intended to replace elastomeric and stainless steel ligatures and has proved to have many advantages over conventional appliance systems. Most of them are related to reduced frictional resistance.

Advantages of metal ligatures are as follows: they do not deteriorate in oral environments, have their shape and strength unchanged, provide less retention of bacterial plaque and are easier to clean in comparison to elastomeric ligatures.^1^ Conversely, the disadvantages are: they are time-consuming and tiresome in terms of placement,^2^^,^ 3 produce variable effects depending on tension, and might occasionally cause discomfort and lesions on soft tissues.^4^^,^ 5 On the other hand, elastomeric ligatures may not provide and maintain full archwire engagement and, in addition, they might increase biofilm retention, thereby hindering good oral hygiene.^6^^,^ 7 ^,^ 8 They also undergo permanent deformation and, thus, force decays with time.^9^ Most important, elastomeric ligatures have shown increased friction in sliding mechanics.^4^^,^ 10

Self-ligating brackets were developed with promises of eliminating ligatures by producing a continuous light force, thereby avoiding frequent appointments for replacement and creating a low friction environment at the bracket-archwire interface, which allows better sliding mechanics and, as a consequence, decreases overall treatment duration.^11^^,^ 12 Nevertheless, whether self-ligating brackets low friction have a clinical effect on faster alignment or space closure, remains under discussion.^13^^,^ 14

For didactic proposes, self-ligating brackets may be divided into two groups, according to the type of ligation: active clip (also known as interactive clip) and passive clip. In an active system, the ligation clip exerts pressure on the archwire against the slot base. The passive self-ligating design uses a closing mechanism that transforms the open slot into a tube.^15^

Different self-ligating brackets showed opening and closure forces that varied among brands as well as among maxillary and mandibular designs of the same brand.^16^ However, all benefits provided by self-ligating brackets can be hindered by a damaged clip, which may affect elasticity and the magnitude of force applied on the archwire, specially regarding active/interactive self-ligating bracket systems. Few studies have been conducted in order to assess clip resistance of self-ligating brackets. Some types of material are more prone to aging due to exposure into the oral cavity by calcification of the adsorbed complexes of ions and proteinaceous matter, which might alter the morphologic, structural and compositional characteristics as well as the mechanical properties of orthodontic alloys and polymers.^15^

In addition to the effects of intraoral aging, another concern might derive from the potential effect of repeated opening and closing movements of self-ligating brackets during the overall term of treatment, in particular for those containing an active/interactive mechanism.

The hypothesis tested in this study was whether an active/interactive self-ligating bracket clip, designed to be flexible and to produce a certain amount of seating force against the archwire, could present changes in stiffness, breakage or permanent deformation during repetitive opening and closure maneuvers.

## MATERIAL AND METHODS

Twenty maxillary incisor ceramic self-ligating In-Ovation C active/interactive brackets (Dentsply GAC International, Bohemia, NY, USA), twenty maxillary incisor Quicklear active/interactive self-ligating brackets (Forestadent, St. Louis, MO, USA), and twenty maxillary incisor WOW active/interactive self-ligating brackets (Hubit, Uiwang-si, Gyeonggy-do, Korea) were used in this study. These specific bracket brands were chosen due to being among the ceramic brackets most used within the Brazilian market, including those that have an active clip and are not made ​​with alloys that present with shape memory.

**Figure 1.**
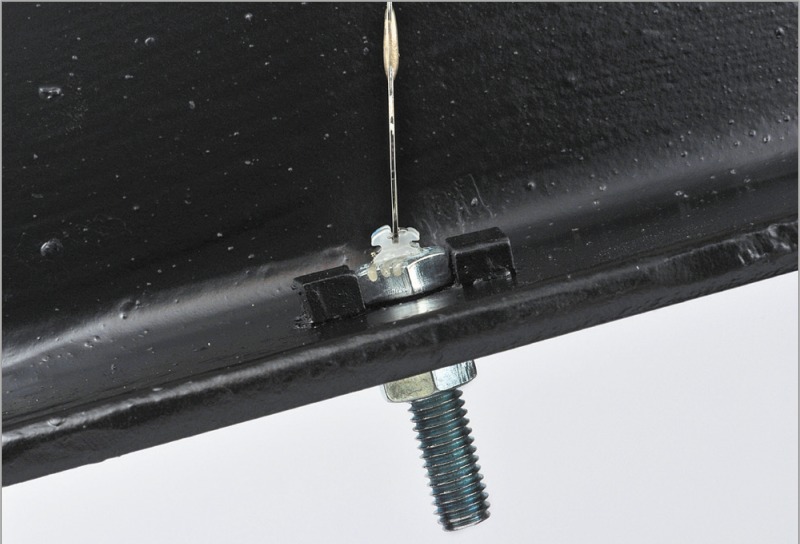
Iron support customized for the tensile test with 12° inclination, so that the bracket slot was parallel to the machine test cell and the stainless steel 0.40 x 0.40 mm wire was engaged.

To assess initial clip stiffness, each bracket clip was assessed by a tensile test performed in an universal testing machine (Emic model DL2000) with a TRD 20 cell (20 Kgf) and 1 mm/min speed. For the tensile test, each sample was fixed to an iron support (Fig 1) 12° inclined (according to the bracket prescription) so that the bracket slot was parallel to the machine test cell, and pulled by a stainless steel 0.40 x 0.40 mm wire. Measurements were performed twice and an average was calculate in order to minimize the possibility of interference or position errors.

Since the universal testing machine is capable of performing various measurements during the tensile test, the resultant force-deflection curves were recorded and divided into three portions. The initial portion, which is parallel to the x axis, corresponds to the sliding of the wire attached to the clip (with no interaction between the clip and the stainless steel wire positioned into the bracket slot); the intermediate portion corresponds to the elastic deformation of the clip (wire-clip interaction); and the final portion corresponds to the locking mechanism of the clip inside the clip notch. In the present study, the line corresponding to the elastic deformation of the clip is defined by the first-order equation *y = ax + b,* and identified by an appropriate software. In this equation, "*y"* stands for force, "*x"* for deformation, "*b"* for the projection of the line in the y axis, and "*a"* for the slope of the line (stiffness). The software uses the coordinates of points on the loading curve to mathematically deduce the stiffness of the clip and compare the initial and final tensile results in order to calculate the plastic deformation of the clip.

After the initial tensile test, the bracket clip was submitted to an opening and closure test of 500 cycles in an automatic device specially designed for this experiment. The automatic device for the opening and closure cycles was constructed over an acrylic table on which four digital servo motors model DS821 (Spektrum, Champaign, IL, USA )were fixed and connected to a controller board and then to a computer interface.

**Figure 2.**
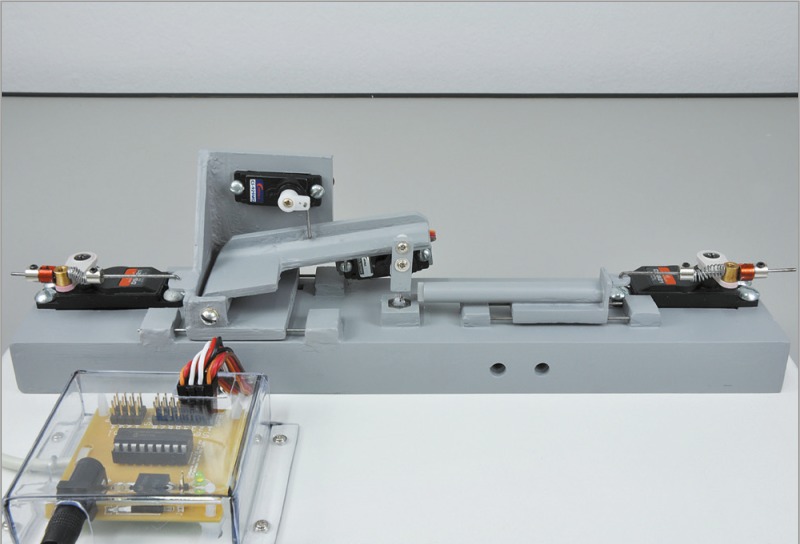
Experiment device configuration. Note the four servo motors at the end of the gray support and the controlling plate for the computer interface.

**Figure 3.**
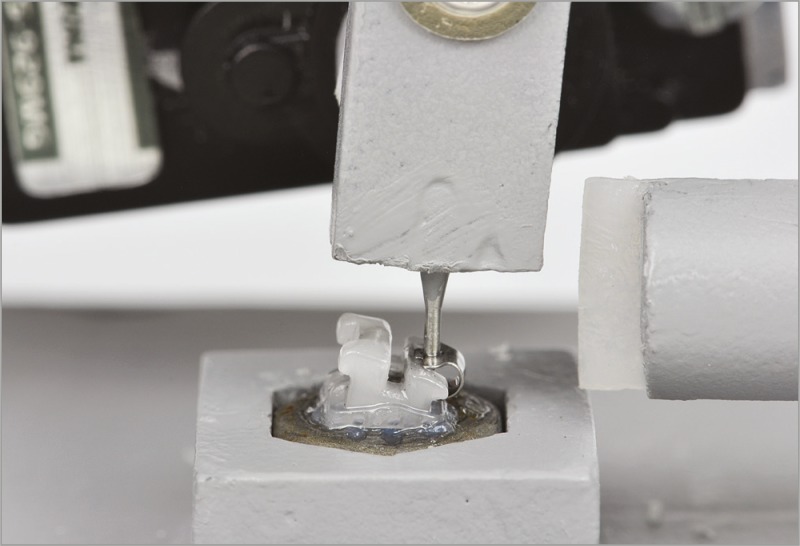
Adapted devices recommended by the manufactures for clip opening and closure.

Two additional components sliding onto 1.25-mm carbon steel rails were tied to the servos on the acrylic table; one containing the active end of the bracket opening instrument, and the other containing the closure device (Fig 2). Both instruments are recommended by the manufacturer (Fig 3) and the force applied was the minimum necessary for the opening and closing of the clip. Speed was calculated and controlled by the software.

To replicate the oral environment, the automatic device and each one of the tested brackets were kept under an acrylic chamber with controlled temperature (37 ^o^C) for 5 minutes before the experiment and during the time necessary to perform the 500 cycles (Fig 3). Additionally, a drop of artificial saliva was placed on top of the clip, so as to mimic the natural lubrication of the oral cavity on the opening/closure bracket mechanism.

Tensile test results reveal that the stiffness of the clip was mathematically deduced throughout the slope of the elastic deformation line. The plastic deformation of the clip was calculated by comparing the displacement of this line between the initial and final tests.

Data of clip stiffness of ceramic orthodontic brackets were subjected to analysis of variance with two criteria for repeated measures and Tukey's test. In order to establish deformation values​​, considering non-normal distribution, we used the non parametric Kruskal-Wallis test. Significance level was set at 5% and all statistical calculations were conducted by means of SPSS 20 (SPSS Inc., Chicago, IL, USA).

## RESULTS

**Figure 4.**
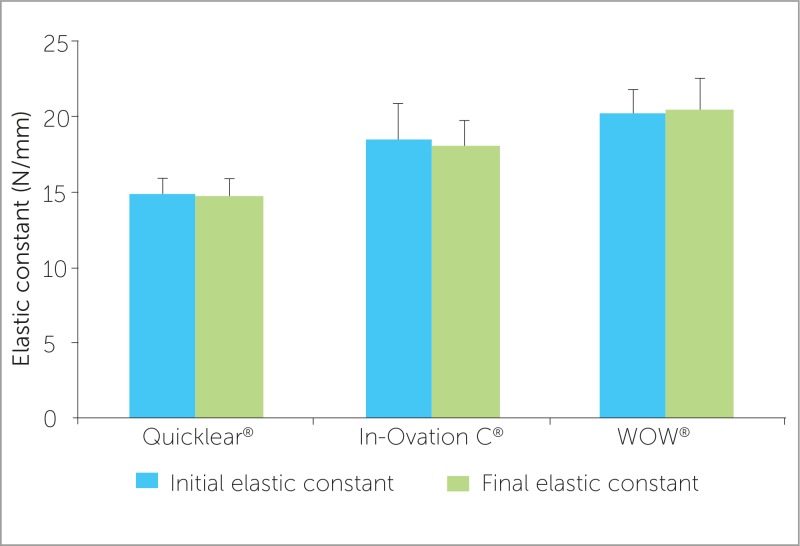
Box plots of measured slope (N/mm) according to the bracket tested and period.

There were significant differences on stiffness among the three types of brackets (*p* = 0.005). The WOW bracket had the highest mean values; whereas the In-Ovation C bracket had intermediate values and the Quicklear bracket had the lowest values, regardless of the 500 opening and closure cycles, as identified by Tukey's test and shown in Figure 4 and Table 1.

**Table 1.** Mean values of elastic constant (standard deviation) of clips, expressed in N/mm, according to bracket brand and time of analysis.

Bracket	Elastic constant		Total mean
	Initial	Final	
Quicklear^®^	14.92 (0.95)	14.91 (0.95)	14.91 (0.92)^A^
In-Ovation C^®^	18.63 (2.29)	18.15 (1.62)	18.39 (1.95)^B^
WOW^®^	20.40 (1.44)	20.71 (1.85)	20.56 (1.62)^C^

* Elastic constant of bracket clips regardless of time. Total mean values followed by different letters suggest significant difference.

Regarding the slope of the force-deflection curve (clip stiffness), data revealed no significant difference among brackets before or after the 500 cycles of opening and closure (*p* = 0.811), as shown in Figure 4 and Table 1.

**Figure 5.**
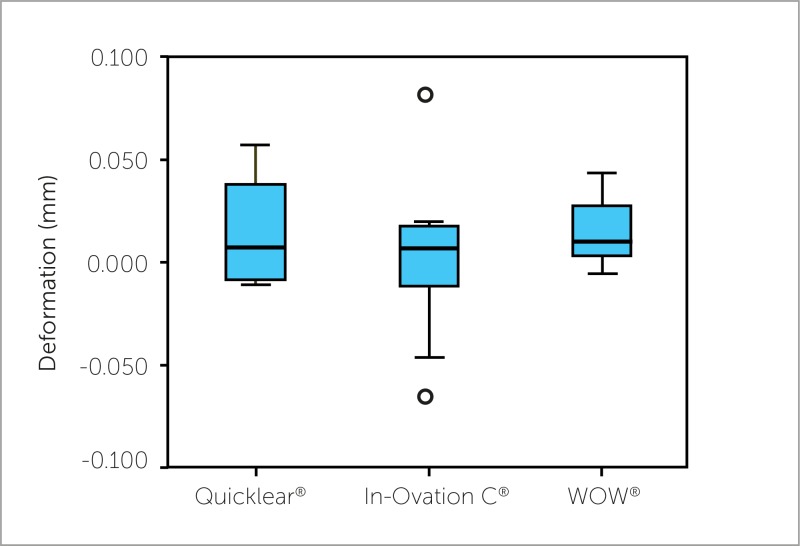
Box plots of plastic deformation range (mm) according to the bracket tested.

The Kruskal-Wallis test showed no significant difference among the values ​​of plastic deformation observed for Quicklear, In-Ovation C and WOW brackets (*p* = 0.205), as shown in Figure 5.

## DISCUSSION

The results yielded herein support the capability of an active/interactive clip, represented in this study by In-Ovation C, Quicklear and WOW brackets, to resist consecutive opening and closure cycles, maintaining the clip integrity with no loss of seating force or clip elasticity and, therefore, keeping ligation secure, fully archwire engagement and resistant to inadvertent loss of ligation.^17^

The benefits of an active clip include early buccolingual alignment, even in smaller archwire dimensions; and achievement of more significant alignment than a passive clip with the same dimension wire, since the existing freedom of the archwire within the bracket slot is lower in active/interactive self-ligating brackets.^18^

The literature about self-ligating bracket clips stiffness and deformation is scarce. Ideally, a clip must be rigid enough so as not to undergo permanent deformation, and flexible enough so as to store, together with the archwire, part of orthodontic forces applied.

In a similar study, Pandis et al^19^ assessed changes in stiffness of retrieved self-ligating brackets, and did not find difference in the force exerted by the clip in SPEED brackets (Speed System Orthodontics, Cambridge, Ontario, Canada) after 15 months of use. Nevertheless, they found extensive relaxation (reduction of nearly 50%) on this force over In-Ovation R brackets (Dentsply GAC International, Bohemia, USA). In our study, absence of permanent deformation or change in stiffness of the clip, for both self-ligating bracket systems used during the tests, may be associated with the use of a standardized instrument, following the manufacturer's instructions, in controlled and ideal conditions regarding the amount and direction of opening and closing forces. Thus, during orthodontic practice, clinicians might manipulate the clip in different conditions, such as archwire/clip interaction in severe crowding with archwire deflection or a more rigid archwire, manipulation of different instruments causing damage to the clip, application of greater force during the opening procedure, presence of masticatory forces in deep bite cases, calculus and plaque formation around the bracket, making the mechanism difficult to open. These factors might contribute to clip breakage and deformation.

Other changes in the self-ligating clip may not only be caused by oxidation of material exposed to the oral environment for a long time, but also by chewing forces and friction due to oral hygiene. The literature is scarce regarding the degradation of the clip during orthodontic treatment; thus, further studies should be conducted on this subject.

The difference in clip stiffness between the two self-ligating bracket systems, in this study and according to results found by Pandis et al,^19^ could be likely due to differences in alloy composition and the manufacturing process of these clips. In order to determine the number of cycles to be applied to this study, a pilot study was performed using 50 cycles and assessment of stiffness and deformation every five cycles. Nevertheless, no significant alteration was found even after 500 cycles of opening and closure movements. Further research should be conducted to test other clinical factors that might compromise clip integrity.

## CONCLUSION

There was no significant change in stiffness or plastic deformation of the clip for both bracket systems used during the standardized controlled test, even after up to 500 cycles of opening and closure movements. There was significant difference on stiffness among the three bracket types: WOW bracket had the highest mean values, In-Ovation C bracket had intermediate values and Quicklear bracket had the lowest values, regardless of the 500 opening and closure cycles.
